# Impact of Eighteen-Year Varied Compliance to Onchocerciasis Treatment with Ivermectin in Sentinel Savannah Agrarian Communities in Kaduna State of Nigeria

**DOI:** 10.5402/2013/960168

**Published:** 2013-08-25

**Authors:** Hudu O. Osue, Helen I. Inabo, Sabo E. Yakubu, Patrick A. Audu, Musa Galadima, Lillian E. Odama, Danjuma Musa, Saleh A. Ado, Mohammed Mamman

**Affiliations:** ^1^Nigeria Institute for Trypanosomiasis Research (NITR), P. M. B. 2077, Kaduna 800001, Nigeria; ^2^Department of Microbiology, Faculty of Science, Ahmadu Bello University (ABU), Zaria, Kaduna State, Nigeria; ^3^Department of Biological Sciences, ABU, Zaria, Kaduna State 810000, Nigeria; ^4^Department of Microbiology, Faculty of Science, Federal University of Technology, Minna, Niger State 920001, Nigeria; ^5^National Institute for Pharmaceutical Research and Development (NIPRD), Idun, Abuja 900102, Nigeria

## Abstract

Baseline and impact assessment data were generated in 1994 (*n* = 532) and 2011 (*n* = 593) from 6 sentinel villages with generalized onchocerciasis. Only volunteers and a cohort (*n* = 445, 75%) were screened at both visits. Each village had received 11 (64.7%) annual treatments and 92.6%, range 88.7–100%, treatment compliance. Overall mean number of treatment was 2.9 ± 1.6 with a range 2.0 ± 1.2–3.3 ± 0.6. Significant decreases in skin microfilaria prevalence from 201 (38%) to 0 (0%), palpable nodule from 77 (15%) to 4 (0.7%), dermal changes from 51 (9.6%) to 2 (0.04%), optic nerve disease from 24 (4.5%) to 4 (2.0%), and onchocercal inducible ocular lesions from 31 (5.8%) to 12 (2.0%) were recorded, *P* < 0.05, (*t*-test of unpaired data). Cases of glaucoma, 8 (1.4%), and blindness, 6 (1.05%), remained unchanged. Visual acuity ≥6/24 in one or both eyes, 198 (33.45%); cataract, 169 (28.5%); pterygium 157 (26.5%); and acute senilis, 165 (27.9%), were significantly increased and positively correlated with increase in age (*R* ^2^ = 0.898 − 0.949). Dissected parous *Simulium damnosum* caught (*n* = 222) were without infective third stage larva. Active onchocerciasis transmission seems halted despite varied compliance to long-term ivermectin treatment. We recommend continued surveillance and targeted treatment of controlled and hypoendemic areas.

## 1. Introduction

Onchocerciasis is a debilitating disease affecting the skin and eye leading to visual impairment and blindness. It is caused by a filarial nematode worm (*Onchocerca volvulus*) transmitted from person to person by the bite of black flies, *Simulium* species. Since the vector breeds in fast flowing rivers and those within the area are exposed to the disease, it is called “river blindness.” It is ranked among the four major preventable causes of blindness in the world, after trachoma, cataract, and glaucoma [1] and the leading cause of blindness in sub-Saharan Africa. Over 20 million people are projected to be infected, 1 million are blind and 70 million at risk of infection worldwide [2]. Nigeria accounts for one third of these estimates. The disease is found in all States of Nigeria with varying degrees of endemicity and severity of clinical manifestations [3–5] Both the savannah type that is associated with severe eye disorders and blindness and the forest type which causes more skin damage are present and responsible for the divergent clinicoepidemiologic picture. One of the major reasons the north of Nigeria is reported to have higher blindness rates than the southern part is owing to the widespread distribution of savannah species of *O. volvulus*. In the south the forest species that cause mostly skin diseases abound as widely reported by some areas with forest-savannah mosaic vegetation known to have both forms [6].

Ivermectin (IVM) or Stromectol (Mectizan) is a drug previously used for veterinary purpose as a broad-spectrum antihelminthes. After IVM was discovered to have microfilacidal activity and proven to be well tolerated, it was patented for human use for mass treatment of onchocerciasis [7, 8]. Ivermectin was adopted in Nigeria in 1992 under the Primary Health Care (PHC) scheme. The control strategy is faced with challenges such as including IVM treatment compliance, emergence of drug resistance, and “poor response” to the drug as variously observed in Ghana. Further, there are numerous constraints that hamper IVM distribution [9–11]. Lately, it was discovered that long-term treatment with IVM not only had effect on microfilaria but also caused death, locomotor paralysis and the loss of fecundity in female adult worms as seen in Latin American countries of Guatemala, Mexico, and Ecuador [12–15]. Early control strategy hitherto depends on larviciding, and erstwhile use of diethylcarbamazine citrate (DEC) and Suramin chemotherapy had proved unsuitable for mass drug administration (MDA). Large-scale nodulectomy was also attempted but without success. These methods failed because of several limitations including insecticidal resistance by the vector, hazard to the environment, and cost given the vast landmass to be covered. In addition, the toxicity of DEC causes “Mazzotti reaction” while Suramin induces serious eye complications [1, 2]. One main objective behind the use of IVM is to break the disease transmission cycle [7]. Added to this, it could assuage deterioration of existing lesions, prevent anterior and posterior segment lesions in the eyes [16–18], or in some cases aggravate them. At this point, we cannot but stress the need for impact assessment of ongoing community directed treatment of onchocerciasis with ivermectin (CDTI) and its long-term effect on parasitological and clinical changes. Previous studies have indicated encouraging potential of breaking the disease transmission cycle in some foci in Latin American countries such as Mexico and Guatemala in which biannual treatment was practiced [19–21]. It will be worthwhile to fully understand the empirical clinical benefits of long-term annual treatment practiced in Nigeria. In the absence of case control placebo studies, baseline data will allow for comparative evaluation and analysis of long-term clinical benefits from IVM treatment. Investigations by others on the impact of 6–8 years of drug control strategy on improvement in health indices such as skin mf, anterior segment lesions, visual impairment, and palpable nodule prevalence have been documented [22, 23]. Equally important is the issue of compliance to treatment, although high rates of this parameter have been reported from different studies carried out across Africa and Latin America [10, 24].

Cross-sectional survey was undertaken in 1994 to collect baseline parasitological, clinical, and serologic data before commencement of mass distribution of ivermectin [25]. Two surveys were conducted ten (10) and eighteen (18) years after, in 2004 and 2011, respectively, to assess the impact of the intervention in the study sentinel villages within the Guinea Savannah ecological zone of Nigeria. This study compared the clinical and parasitological changes 18 years after treatment over the baseline data.

## 2. Methodologies

### 2.1. Study Area/Population

The study area consisted of villages (Bomjock, Gantan, Gidan Tama, Kurmin Gwarza, Sabo Gantan, and Unguwar Shaho) in Kachia Local Government Area (LGA) of Kaduna State, Nigeria. They are located within 9° 37–9° 45′ N and 7° 44–7°48′ E. The type of vegetation is reminiscent of wooded-shrub grassland with 80–90% farmland [26] and lies within the subhumid climatic zone. Bomjock is farther more in the hinterland and is a small non-autonomous farming settlement under K. G. with which they share ancestral affinity. Sabon Gantan has history of resettlement in a once abandoned site by inhabitants of Gantan with which they share ancestral lineage. Bomjock, S. Gantan, and Gantan are situated close to one of the tributaries of River Gurara called River Gantan. Both Gantan and K.G. are 3 Km apart, both are accessible by a federal highway leading to Nasarawa State, while Ungwar Shaho and Gidan Tama are relatively close to the tributary of River Gantan but are far away from the highway.

**Figure 1:**
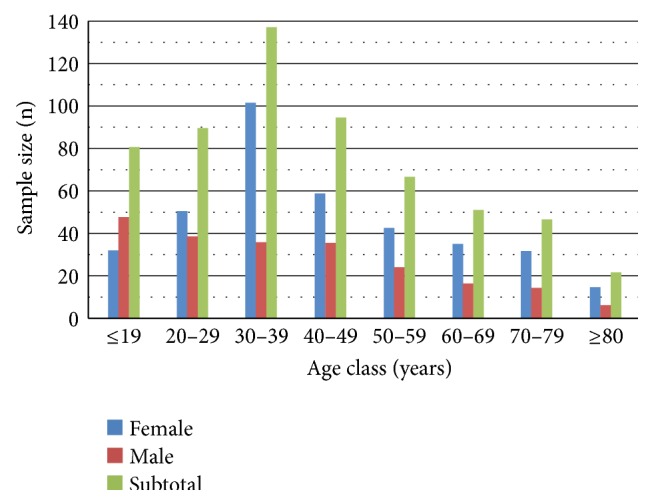
Age class distribution of sample population.

Baseline sample population, *n* = 532 (male: 297 and female: 235), and impact assessment sample population, *n* = 593 (male: 208 and female: 385), volunteered to participate in the two studies. The overall study populations of 900 and 1230 are based on figures provided by the community directed distributors (CDDs) in the sentinel villages. The mean ages of the sample populations were 42 ± 19, with a range 5–90 yrs old. Age class distribution of impact assessment study population is given in Figure 1. River Gurara has a network of tributaries and rivulets that extend to the study area. The people are mostly peasant farmers who grow ginger and soya bean as cash crop, maize, guinea corn, groundnut, and yam as staple food crop. Only adults (aged 15 years and above) were examined during baseline studies. The villagers were briefed in their dialect on the purpose and method of the study through a member of the team who speaks the dialect assisted by Primary Health Care (PHC) staff of the Local Government Health Department assigned to the team. Only persons who freely consented to participate were enlisted for screening. The research protocol was approved and funded by the Nigerian Institute for Trypanosomiasis Research (NITR) which has the national mandate to carry out research into African trypanosomiasis and onchocerciasis. Ethical clearance was obtained from the Kaduna State Ministry of Health for the impact assessment.

### 2.2. Bioclinical Data

Each participant was clerked to obtain vital information on gender, occupation, years of residence, number/dosage of ivermectin taken, and any adverse effect observed after treatment.

### 2.3. Geospatial and Entomology Data

A handheld Geographic Position System (GPS), eTrex, Legend, Garmin, was used to capture the coordinates of the study sites and black fly locations. Breeding sites were prospected for along the rivers, streams and rivulets at the villages during impact assessment study. Three adult males were used as human bait to catch flies in the morning for three days at the Gurara Village, a well-known black fly breeding sites [27]. The black flies caught were separated into nulliparous and parous groups; they were preserved in 80% ethanol. Only the parous flies were dissected under a Wilde dissecting microscope at x4 magnification to determine infection rate. In addition, the flies were broadly characterized into savannah and forest types depending on the color of the wing turf.

### 2.4. Parasitology

Skin snips were obtained from both iliac crests of each person using Holth corneoscleral biopsy punch with 1-2 mm bite. After the sampling of each patient, the instruments were washed sequentially with chlorhexidine, bleach, distilled water, and alcohol, and then air-dried. The skin specimens were transferred to a microtiter plate containing 100 *μ*L normal saline, incubated for 24 hours at room temperature, and removed, and the fluid in each original well was fixed by adding two drops of 40% formosaline as described by Emukah et al. [22]. Emerged microfilariae, which served as infection intensity, were enumerated under an inverted microscope at 40x using a tally counter. Blood samples collected from a subpopulation (*n* = 250) were used to prepare thin blood films that were stained with Giemsa stain and examined under ×40 objective microscope for microfilaria.

### 2.5. Clinical Examination

Each individual was subjected to medical and ophthalmic examinations. All parts of the body of participants were screened by a Principal Rural Health Superintendent in 1994 and a medical doctor in 2011 for dermatological lesions and palpable nodules as far as decency permits especially with females. Eye examinations were performed by an ophthalmic nurse and a medical doctor. Both staff had been trained for their specific roles and well experienced in the field. The skin changes sought included papular onchodermatitis, hypopigmentation or “leopard skin” and skin atrophy (only in those below 50 years of age), edema, hanging groin, and lichenified skin [28]. Swinging torch loop and ophthalmoscope were used to examine the anterior and posterior segment lesions. Visual acuity (VA) of each eye was measured using illiterate Snellen E-chart and the field of view was assessed based on the ability to see fingers at four quadrants. The visual status was categorized as normal if VA = 6/18 or better in either eye and visual impairment if VA is between 6/24 and 3/60 or in the case of the inability to count fingers in conformity with the World Health Organization guidelines [1]. The various onchocercal inducible eye lesions described by Dadzie et al. [29] and Whithworth et al. [16, 17] were adopted. They include punctate keratitis, sclerosing keratitis, iritis, cataract, optic nerve (totally white nonglaucomatous optic disc), vascular sheathing, and chorioretinitis. Glaucoma or intraocular pressures (IOP) were screened for using observable cupping of optic disc in the absence of applanation tonometry.

### 2.6. Annual Treatment Coverage and Sample Population Compliance Rate

Information on village treatment dose and individual compliance rates were obtained from the CDDs and participants. The village mean annual treatment coverage (ATC) was based on the number of times drugs were distributed divided by the expected total treatment from 1994 to 2011 (17) multiplied by 100. The mean sample population compliance was derived from sum of number of treatment received by participants divided by sample population.

### 2.7. Data Analysis

Data were double fed into personal computer to ensure error free entry. The mean and percentages were calculated using Microsoft Excel. Geometric mean microfilarial count or community mf count for those aged 20 and over was calculated using the formula **e** ^Σlog(count+1)/n^ [30]. Descriptive statistics and tabular data presentations were made. Those with microfilaria >0 are regarded positive.

## 3. Results

### 3.1. GPS and Entomology Data

Out of the 1222 black flies caught at Gurara Village located at 9°40′13.18′′N and 7°52′58.04′′E comprised of 1000 nulliparous and 200 parous (engorged) flies. Gurara Village is about 3 Km to Bomjock, 8 Km to Gantan, and 12 Km to U/Shaho and G. Tama. Twenty-two (22) flies were caught at Unguwar Shaho within the vicinity of the Primary School (9°42′46.13′′N and 7°55′40.20′′E) used as screening center. None of the engorged flies were infected. All the 222 parous black flies had grey wing turf.

### 3.2. Parasitology

The baseline overall prevalence of onchocerciasis based on emergence of mf from either right or left iliac crest skin snips was 37.9%. This varied between villages (22% to 72%) with mean microfilarial density of 17.7 mf per skin snip. The community microfilarial load (CMFL) or the geometric mean microfilarial count for those aged 20 years and over was 1.5 mf per skin snip. The higher prevalence in men (41.8%) compared to women (34.7%) was also found for all villages individually, except in Kurmin Gwaza (Table 1). Onchocercal nodules were palpable in 77 (14.5%) with males 33 (13.9%) and females 44 (15%) with the number of nodules ranging from 0 to 8 per person. Over 60% nodules were located in the pelvic region. The CMFL and skin microfilarial and palpable nodule prevalence increased with increase in age except that there was a decrease in frequency in the 50–59-year age group. The ≥60 yrs group had 5.4, 36 (57.1%), and 27 (42.9%), respectively. At posttreatment, the skin and blood microfilaria prevalence was 0 (0%) and the number of nodules significantly decreased to 4 (0.7%) as shown on Table 2.

### 3.3. Onchocercal Skin Clinical Signs and Symptom

At pretreatment, the complaint of itching was a commonplace among the villagers; hence it was not scored. Twenty-four persons (4.5%), 4 (1.7%) males and 20 (6.8%) females, did not differ from 22 (3.7%) with visible scratch marks. Fifty-one (51) (9.6%) and 0 (0%) presented with skin clinical signs that included papular onchodermatitis, 37 (7%); leopard skin, 10 (1.9%); skin atrophy, 12 (2.3%); and 6 (1.1%) cases of hernia. Prevalence was more in females than males with 26 (8.8%) and 11 (4.6%) manifesting papular onchodermatitis, 6 (2%) and 4 (1.7%) skin atrophy, and 5 (1.7%) and 1 (0.4%) hernia, respectively (Table 3). Strikingly, the peak frequencies for both skin and eye manifestations were recorded in the 50–59-year group with prevalence of 36 (57.1%) and 27 (42.9%).

On the contrary, there was significant reduction (*P* < 0.01) to 2 (0.4%) in skin clinical changes at post-treatment. Other notable observations of public health importance were guinea worm infection and bilateral lymphedema possibly by *Wuchereria bancrofti* in a 65-year-old and 72-year-old males at Sabon Gantan. There were 5 (0.84%) cases of corneal scars, allergic ophthalmitis 2 (0.4%), and trachoma (*n* = 1). Midge infestation in 2 adult males was seen in Unguwar Shaho Village.

### 3.4. Annual Ivermectin Treatment Coverage and Compliance Rates

**Figure 2:**
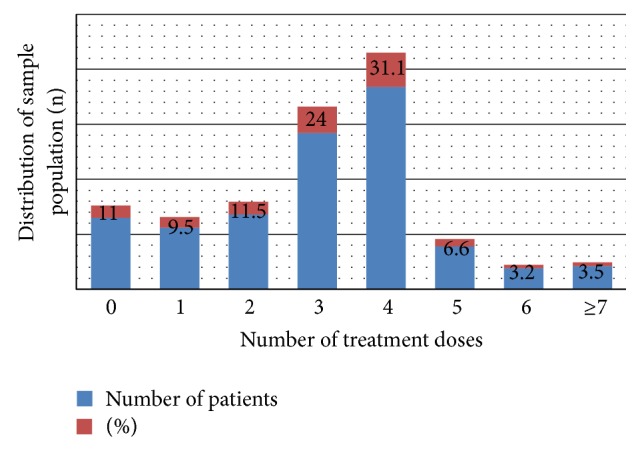
Doses of ivermectin taken by participants from 1994 to 2011.

The study population was apparently stable as migration into the village within the period by residences of ≤10 years ranges between 5.3 and 14.1% as shown in Table 4. All the villages had 11(64.7%) annual treatment coverage. The sample population treatment compliance rate varied from 85.6 to 100% with mean dose of 2.9 ± 1.6 (with a range 2.1 ± 1.7–3.4 ± 1.6). Those who received at least treatment once were 89.0%.  Figure 2 shows the distribution of annual number of ivermectin treatment doses taken from inception comprising of those that did not receive treatment 65 (11.0%) and those who received 1 to ≥7 treatment doses. The participants who received 4 treatment doses were 180 (30.4%) followed by those who received 3 treatments 140 (23.6%).

### 3.5. Eye Clinical Manifestations

Table 6 shows the age specific prevalence of individuals with poor visual acuity and ocular lesions. All those manifesting restricted vision had optic nerve disease. The incidence of onchocercal inducible eye lesions was recorded in 31 (5.8%) persons. Twenty cases (64.5%) had poor field of view representing 3.6% prevalence rate. Cases of visual impairment and blindness in one eye were 4 (0.8%) and 5 (0.9%), while those affecting both eyes were 1 (0.2%) and 2 (0.4%), respectively. Incidence of optic nerve disease was 24 (4.5%) with male having 12 (5.1%) and female 12 (4.1%): others were glaucoma 8 (0.6%), onchocercal sclerosing keratitis (0.6%), and cataract (0.8%). A case of punctate keratitis in a 16-year-old boy was noticed. Three (3) cases of unilocular and one (1) case of bilateral blindness due to optic nerve atrophy and glaucoma, respectively, were also observed.

Ten persons (1.9%) had nononchocercal related ocular complications. They included a case of bilateral blindness due to trachoma which manifested as corneal opacity with pannus, a case both of poor refractive media and corneal opacity due to ulcer or measles, unilocular and bilateral pterygium, and corneal or macular scars. Others were two cases of corneal edema due to trachoma and *Leucoma* due to measles. A woman had lepromatous leprosy and another suspected to be a borderline case leprosy were among those reporting for screening. The former was not screened, but the latter was skinsnip negative.

**Figure 3:**
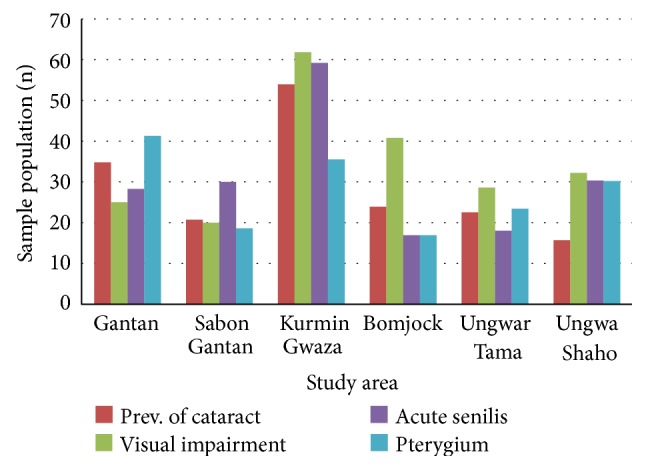
Post-treatment village prevalence of ocular clinical manifestations and visual impairment.

**Figure 4:**
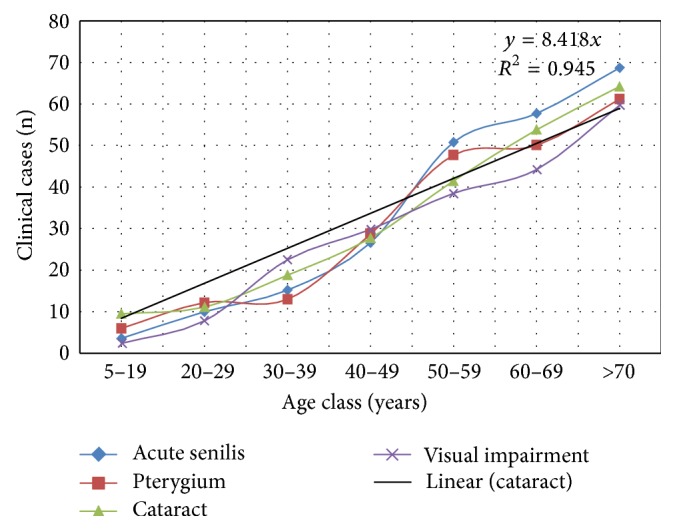
Age class prevalence of ocular manifestations

**Figure 5:**
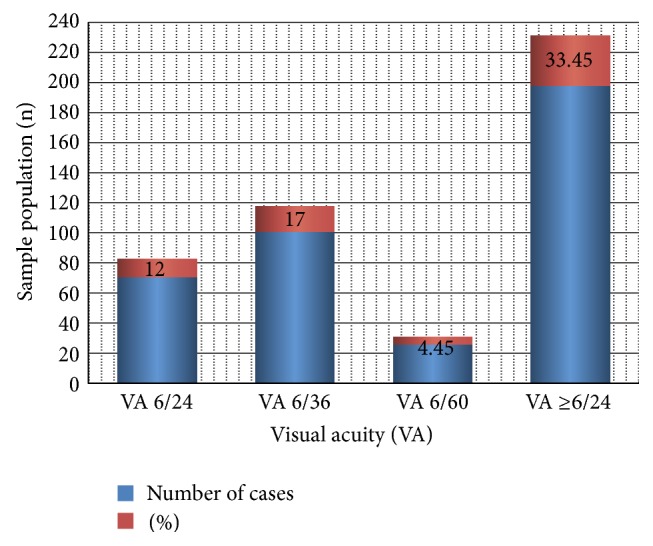
Cases of visual acuity measured with Snellen's illiterate E-chart.

Post-treatment impact study showed there was a significant reduction (*t*-test, *P* < 0.01) in prevalence of optic nerve disease to 4 (0.7%) and skin clinical manifestations to 2 (0.4%). There was no change in cases of glaucoma 8 (1.4%) and blindness 6 (0.9%), and no new blind case was recorded (Table 5). Among the villages frequencies of visual impairment were varied; poor visual acuity, cataract, and acute senilis were highest in K. Gwaza, while Gantan had the highest rate of pterygium (Figure 3). Those with two or three ocular manifestations in an individual were as follows: cataract and acute senilis 114 (32.0%), the highest combination followed by A. senilis and pterygium 89 (25.0%), cataract and pterygium 88 (24.0%), and those with three lesion 69 (19.0%). Prevalence of visual impairment and the three other ocular clinical manifestations were strongly and positively associated with increase in age (Figure 4) with *R* ^2^ values ranging from 0.898–0.949. Generally, the number of visual impairment increased remarkably to 168 (32.3%).  Figure 5 showed that those with VA ≥ 6/24 were 198 (33.45%) with 26 (4.9%) with VA of 6/60 that were severely visually impaired among the older age group.

## 4. Discussion

Impact of 18 years of IVM treatment of onchocerciasis was assessed in six sentinel villages. Pre-treatment data and post-treatment parasitological and clinical data were compared. The study villages were georeferenced with spatial and temporal (attribute) data captured; there was no baseline entomological study. The fact that no breeding site was located during impact assessment undertaken at the peak of raining season may be due to overflooding and the breeding sites have been submerged. The study area is no doubt within previously active onchocerciasis endemic savannah foci of Nigeria. The flies caught in three days showed that high fly population density will be a sustained source of ensuring onchocerciasis transmission and its attendant severity of biting nuisance. Absence of no infection in flies has been acclaimed as alternative means of establishing a break in disease transmission. This study has provided the need to intercept man-simuliid vector contact at the study area as suggested by Adeleke et al. [31]. The persistence presence of huge fly population is inimical to full utilization of the highly needed arable fertile riverine alluvial land for agricultural crop farming. There was a strong possibility of peridomestic transmission among the ginger farmers in Bomjock and Sabo Gantan, both situated at the upper course of the river. Vector-host contact was strongly suggested by catching of flies at the vicinity of the primary school used as screening centre at Unguwar Shaho. River Gurara and its tributaries and rivulets are undoubtedly the major and minor breeding sites for the vector black flies. Hence, agricultural productive capacity of these farmers may be adversely affected as noted by Ufamadu et al., [32] among rice farming communities elsewhere. It was the growing need for more fertile farmlands that made inhabitants of Sabo Gantan to resettle in abandoned location and Bomjock settled in virgin lands. Unlike the others, these two villages complained of high fly biting activities, even within the vicinity of their houses. This may be responsible for their baseline meso- and hyperendemicity, respectively. This could be attributed to their proximity to a breeding site. It is strongly believed that the high population of black flies along River Gurara and its tributaries may, in addition to the disease transmission and the painful bites of the insect, be intolerable nuisance that could sometimes lead to blood loss. Secondly, the flies could serve as vehicles for viruses, bacteria, protozoa, and nematodes which may carry on their bodies or exist in the environment as suggested by Ubachukwu, [33], Usip et al. [34], and Adeleke et al. [31]. This has necessitated the black fly control to remain an important public health concern in Gurara onchocerciasis endemic focus in particular and other areas infested with the black flies in general.

Baseline result had shown that the generalized type of disease was present in the study area. Some persons with low skin mf load and prepatent infections may have invariably gone undetected. These are among the factors known to hamper the sensitivity of skin snip for microfilarial detection, which improves with the number of snips [35]. Intrigued by that fact, there are persons with large numbers of skin mf with no overt clinical manifestations. Paradoxically, subjects with no skin mf and no palpable nodule had onchocerciasis related dermal and/or ocular signs. A similar finding has been documented for filariasis, including onchocerciasis [36], which is in contrast to commonly observed association between severe infection intensity and clinical manifestations [4, 16, 17]. It is assumed that clinical signs may not be direct consequence of the parasite but may be indirectly due to host responses to the infection. Prevalence rate, skin mf load, CMFL, and clinical signs increased with age irrespective of sex. In the study villages, there were no sociocultural and religious limitations on female's involvement in farming and related activities. Women do a lot of farming activities in addition to the core duties of fetching firewood and water; they also go to river to wash their clothes. Traditionally, males play more active roles and are involved in farming to greater extent than their female counterparts. Therefore, males are at marginally higher risk of exposure to infection than females. This explains the slight higher frequency of the disease in males.

Whether the significant higher prevalence of skin manifestations among females compared to males is due to gender-related physiologic, hormonal, and immunological differences can only be speculated. Inherent factors like sex, race, and genetics are thought to influence the pathology of the disease [37]. The equal prevalence of ocular involvement in both sexes contradicts the averred higher frequency and severity of ocular lesions in males than in females [16, 17, 38]. Therefore, development of eye pathology may not be influenced by gender in the study area. Expectedly, more men will be blind in areas where women are restricted from farming and carrying out other related activities as already mentioned.

Optic nerve disease was ranked highest in frequency (4.5%) among the ocular lesions detected in sampled population. In view of the above, the enlistment of the communities in the nationwide mass treatment coverage in 1994 was timely. It was envisaged that the huge reservoir of parasites will be cleared [16, 17, 19, 29]. As suggested by Abiose et al., [38], this study confirmed that many of those with 10 or more mf per skin snip at risk have been prevented from developing optic nerve disease. Unexpectedly, high prevalence rates of cataract, pterygium, and acute senilis were observed after long-term posttreatment. Cataract is associated with visual impairment as reported by Nmorsi et al. [39] despite ivermectin treatment. This was corroborated by data from this study with overall prevalence of cataract 169 (28.5%), A. senilis 167 (27.9%), and visual impairment 157 (26.5%).

It was observed from the Kachia LGA Onchocerciasis Control Coordinator and CDDs that combined mass treatment with ivermectin and Albendazole in the study areas started in 2005. Coendemicity of onchocerciasis, lymphatic filariasis, malaria and guinea worm had coexisted in the study area. The effect of combining the two drugs and IVM alone for treatment annually or biannually potentiates clearing of microfilaria and the reduction in their release by female adult worms of *O. volvulus* [40]. How this may have played a role in the outcome of this study cannot be fully deduced. Presence of a case of guinea worm during this study clearly indicates that eradication of the disease faces challenges by identifying an isolated case reported in this study. The study area is a coterminal falariasis and guinea worm focus.

In this paper, we have shown that the sentinel villages were active transmission sites of onchocerciasis with emerging clinical trend at the time of baseline survey. The disease dynamics has changed with a possible break in active transmission and the development of new lesions has been halted. This is in agreement with similar findings in two different foci within the same state by Tekle et al. [41]. They reported that the median prevalence pre-treatment infection levels of 52 per cent were reduced to 0 per cent 20 years after treatment. These outcomes were achieved despite the varied rate of annual community coverage and individual treatment compliance rates in the study areas. This is in conformity with what reported by Emukah et al. [22], which indicated that differences in community coverage did not appear to influence the benefit from treatment of individual residents. It is important to note that Bomjock despite being hyperendemic with the least number of doses 8 (44.4%) and mean treatment compliance had (2.0 ± 1.2) had zero skin mf prevalence. Despite having received one treatment dose in 1994 as at 2003, the sample population showed a significant decline in skin mf prevalence and palpable nodule (Osue et al., unpublished data) that accounted for the highest rate among the villages. The observed improvement in IVM distribution was possibly influenced by the 2003 impact assessment study. 

Although the risks of skin and eye lesions have been mitigated, important factors like cataract which is responsible for visual impairment remain a serious issue in the study area. Evidence of itching constitutes a disturbing factor that will somewhat diminish man-hour loss available for agricultural labor in black fly infested areas. These long-term longitudinal studies have provided practical evidence for empirical benefits of annual IVM treatment in breaking disease transmission dynamics and prevented development of new onchocerciasis-induced skin and eye clinical lesions. Where control has been achieved as in OCP operational areas, the need for continued surveillance cannot be underscored [42]. The low rate of clinical cases may be in part due to the fact that some of the early patients who had died or were not present for reevaluation had no significant influence on the outcome of this study. Noteworthy, there was no new case of the disease either.

Comparing the impact of medium term (6–8 years) annual and biannual drug treatments have been reported to culminate in reducing transmission, irreversible effect on adult worms, prevalence of skin mf, and clinical lesions [24, 43–45]. The ten-year post-treatment assessment of the study area confirmed reduction in these parameters (Osue et al., unpublished data). Therefore, the estimated 15–25 years for mass drug administration using IVM in West Africa showing promise of eliminating the disease [18, 46] may need to be reviewed to meet with reality of using targeted drug distribution proposed by [47]. It is obvious that we have to contend with the problem associated with logistic distribution and apathy on the part of the people to the control strategy of MDA. We posit the need to adopt the bi-annual treatment based on its proven impact in Latin Amecrican countries in achieving a break in onchocerciasis transmission within a short term [20, 21]. In the event of availability of alternative drugs calls for the revaluation of the on-going CDTI program in Nigeria, in particular West Africa and sub-Saharan Africa in general. Hence, biannual treatment of targeted individuals in endemic communities appeared to be a promising futuristic approach that will suffice using IVM and any one of the potential candidate macrofilaricides presently at various stages of clinical trials is eventually approved.

## Figures and Tables

**Table 1:** Pretreatment village specific prevalence and intensity of *O. volvulus* skin microfilariae and palpable nodules.

Village	*n*	Mf no. +ve (%)	Mf per snip	Nodule no. +ve (%)
Sabon Gantan	96	50 (52.1)	25.5	27 (28.1)
Kurmin Gwarza	89	32 (36.0)	9.8	13 (14.6)
Bomjock	50	36 (72.0)	20.4	11 (22.0)
Gidan Tama	100	37 (37.0)	15.9	9 (9.0)
Ungwar Shaho	100	22 (22)	16.1	7 (7.0)
Gantan	96	24 (25.0)	11.8	9 (10.4)

Total	531	201 (37.9)	17.7	77 (14.5)

**Table 2:** Pretreatment age specific prevalence and intensity of microfilariae and palpable nodules in study population.

Age group	*N*	Mf^a^no. (%) +ve	GM^b^	Nodule No. (%) +ve
15–19 yrs	90	12 (11.1)	1.3	1 (1.1)
20–29 yrs	151^c^	56 (37.1)	2.7	7 (6.0)
30–39 yrs	108	46 (45.4)	3.8	13 (13.0)
40–49 yrs	67	33 (49.3)	4.5	18 (26.9)
50–59 yrs	52	17 (32.7)	2.6	17 (32.7)
≥60 yrs	63	36 (57.1)	5.4	27 (42.9)

^a^Mf: microfilariae, ^b^GM: geometric mean (calculated as **e** ^Σlog(count + 1)/*n*)^, and ^c^one person refused to be skin snipped.

**Table 3:** Participants years of residences at post-treatment.

S/no	Village	≤10 years	≥11 years	Sample population
(1)	Gantan	10 (10.9%)	82 (89.1%)	92 (15.5%)
(2)	Sabon Gantan	16 (11.4%)	124 (88.6%)	140 (23.6%)
(3)	Kurmin Gwaza	4 (5.3%)	72 (94.7%)	76 (12.8%)
(4)	Bomjock	10 (14.1%)	61 (85.9%)	71 (12.0%)
(5)	Gidan Tama	17 (13.5%)	109 (85.5%)	126 (21.3%)
(6)	Ungwar Shaho	10 (11.5%)	77 (89.5%)	87 (14.7%)

	Total	67 (11.3%)	525 (88.7%)	592 (100%)

**Table 4:** Eighteen years of ivermectin post-treatment coverage of the study area.

S/No.	Study area	Population treatment coverage (%)	Total village treatment doses	Mean dose
(1)	Gantan	91.3	11	3.2 ± 2.8
(2)	Sabon Gantan	100	11	3.3 ± 0.6
(3)	Kurmin Gwaza	85.6	11	2.1 ± 1.7
(4)	Bomjock	88.7	8	2.0 ± 1.2
(5)	Gidan Tama	96	11	3.1 ± 1.7
(6)	Ungwar Shaho	94.2	11	3.4 ± 1.6
(7)	Total	92.6	11	2.9 ± 1.6

**Table 5:** Changes in skin microfilaria and clinical signs.

S/No.	Parameter	Post-control data (observed, *n* = 592)	Baseline data (expected, *n* = 531)	Impact of treatment (%)	Statistics test
(1)	Skin mf prevalence	0	144 (27.0%)	−100	*t*-test of unpaired data *P* < 0.01
(2)	Nodule positive	4 (0.7%)	77 (14.4%)	−95.1	-do-
(3)	Pruritus (scratch marks)	22 (3.7%)	24 (4.5%)	−17.8	*P* > 0.05
(4)	Skin clinical manifestations	2 (0.4%)	51 (9.1%)	−96.2	-do-
(6)	Optic nerve disease	4 (0.7%)	24 (4.5%)	−77.8	*P* < 0.05
(7)	Inducible eye lesions	12 (2.0%)	31 (5.8%)	−65.5%	*P* < 0.05
(8)	Blindness (no perception of light)	6	6	0	Unchanged, no new case detected

**Table 6:** Prevalence of *onchocercal* related ocular clinical manifestations in study population.

S/no.	Study area	Sample size (*n*)	Acute senilis	Pterygium	Glaucoma	Cataract
(1)	Gantan	92	26 (28.3%)	38 (41.3%)	0	32 (34.8%)
(2)	Sabon Gantan	140	42 (30.0%)	26 (18.6%)	0	29 (20.7%)
(3)	Kurmin Gwaza	76	45(59.2%)	27 (35.5%)	3 (3.9%)	41 (53.9%)
(4)	Bomjock	71	12 (16.9%)	12 (16.9%)	4 (5.6%)	17 (23.9%)
(5)	Ungwar Tama	126	20 (18.0%)	26 (23.4%)	0	25 (22.5%)
(6)	Ungwa Shaho	87	20 (30.3%)	28 (42.4%)	1 (1.5%)	25 (37.9%)

	Total	592	165 (27.9%)	157 (26.5%)	8 (1.4%)	169 (28.5%)
